# Palliative laparoscopic resection of renal cell carcinoma metastatic to the stomach: report of a case

**DOI:** 10.1186/1477-7819-12-394

**Published:** 2014-12-23

**Authors:** Thiago Nogueira Costa, Flavio Roberto Takeda, Ulysses Ribeiro, Ivan Cecconello

**Affiliations:** Division of Digestive Surgery - Department of Gastroenterology, São Paulo State Cancer Institute - ICESP-HCFMUSP, University of Sao Paulo School of Medicine, Avenida Doutor Enéas de Carvalho, 255, São Paulo, SP Brazil

**Keywords:** laparoscopic wedge resection, stomach, renal cell carcinoma, metastasis, palliative

## Abstract

The most common sites of metastases in renal cell carcinoma (RCC) are lung and bone. However, unusual sites, including the stomach, are characteristic of RCC.

This article presents a case of a metastatic RCC (lung and liver) with a symptomatic gastric metastasis treated by a laparoscopic wedge resection (LWR).

A 66-year-old woman, diagnosed with RCC underwent a right nephrectomy. During her follow-up, an upper gastrointestinal (GI) endoscopy showed an ulcerated lesion at the stomach. A biopsy of the specimen revealed metastatic RCC. The patient underwent a palliative LWR and was discharged home 8 days after surgery.

Therefore, LWR is a relatively simple technique with the advantages of minimal invasive access in the treatment of palliative cases.

## Background

Renal cancer is the 12th leading malignant condition among women and the 7th among men in the United States and accounts for 2.6% of all cancers. The median survival of renal cell carcinoma (RCC) in metastatic disease is 13 months. A quarter of patients with RCC present with advanced disease, including locally invasive or metastatic cancers. Moreover, a third of patients undergoing resection will have recurrence [1].

The two most common sites of metastases in RCC are lung (up to 60% of patients) and bone (up to 40% of metastatic patients). However, unusual sites of metastasis are typical of RCC and include the thyroid, pancreas, skeletal muscle, the skin or underlying soft tissue and virtually any organ. Among these metastatic sites, metastasis to the stomach is quite rare, even in autopsy studies [2–10].

Other metastases to the stomach are rare (ranging from 0.2 to 0.7), and they have been reported to result mainly from breast cancers, lung cancers, and melanoma [2, 3]. The main symptoms of these metastases are bleeding, anemia, epigastric pain and outlet obstruction [4–8]. In addition, according to Green *et al*. [11], chemotherapy in patients with unrecognized stomach metastases may result in tumor necrosis followed by perforation of the stomach and rapid death. The treatment options could be embolization, epinephrine injection, endoscopic resection or surgery [12–17], even in palliative patients [7].

Herein, we present and discuss a case of a metastatic RCC (lung and liver) with a symptomatic gastric metastasis treated by a laparoscopic wedge resection (LWR). Our aim is to show a rare case of metastasis to the stomach and highlight the use of a minimal invasive technique for palliative use.

## Case presentation

A 66-year-old woman who was diagnosed with renal cell carcinoma (RCC) underwent a right nephrectomy in January 2006; TNM classification was pT3a pN0 pMx. In June 2008, she presented with a metachronous lung metastasis without any symptoms (only seen in routine image studies), and 5 months later she showed various metastases to the liver, maintaining no symptoms.

**Figure 1:**
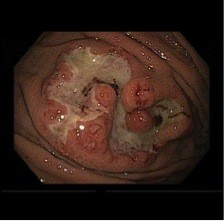
**Upper GI Endoscopy.** Gastric endoscopy confirmed an ulcerated lesion.

By December 2011, she presented symptomatic anemia (hemoglobin level was 5.7 g/dl). An upper gastrointestinal (GI) endoscopy showed an ulcerated and friable lesion, covered with fibrin, which measured 25 mm at the greatest diameter and was located at the greater curvature of the gastric proximal body (Figure 1). The specimen’s biopsy histology and immunohistochemistry revealed similar patterns as the RCC resected from the patient years previous.

**Figure 2:**
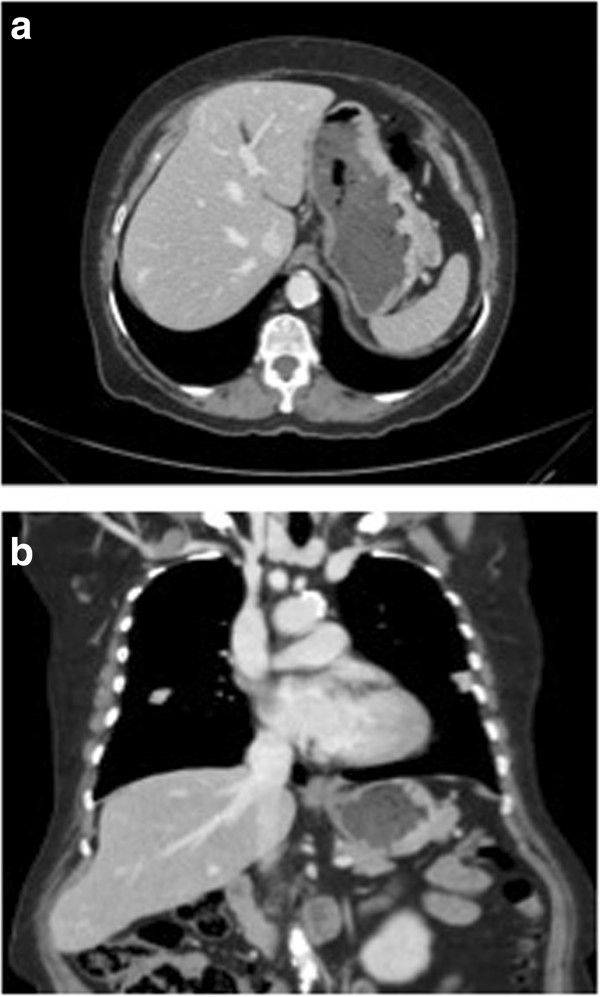
**Tomography findings.** Tomography images showing an exofitic mass in the greater curvature of the stomach. **a**: axial **b**: coronal.

Computed tomography showed an exophytic gastric mass in the greater curvature of the stomach (Figure 2a,b).

This patient was hospitalized because of her symptoms. During her stay at the hospital, gastrointestinal bleeding persisted, necessitating a transfusion of six blood units.

**Figure 3:**
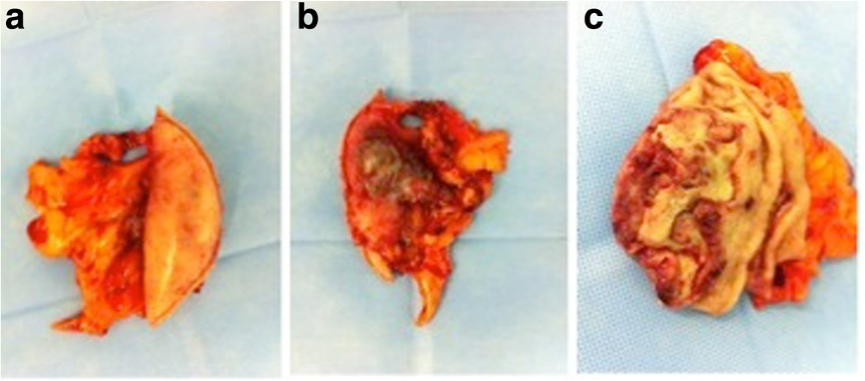
**Macroscopic view.** Macroscopic appearance of resected specimen. **a**: posterior view **b**: anterior view **c**: open view.

Because of the persisting symptoms, she underwent a palliative laparoscopic wedge resection with an auxiliary incision (3 cm) in the beginning of February 2012.The product of resection was a gastric segment measuring 8.0 × 5.8 × 2.3 cm, and the lesion, which measured 5.3 × 2.2 × 2.3 cm, was ulcerated and covered with fibrin (Figure 3a-c). The histopathological study of the specimen showed margins free of tumor and no perineural invasion. Immunohistochemistry indicated positive Cytokeratin AE1/3, positive CD10, positive CK 7 and positive vimentin.

After surgery, which lasted 40 minutes, the patient was sent to the intensive care unit (ICU) where she stayed for one day. During her hospital stay, she developed a mild pneumonia treated with antibiotic therapy (tazobactan + piperacillin) for 7 days.

She was discharged home 8 days after surgery and returned 1 week later at the outpatient clinic without any symptoms.

## Conclusions

Gastric metastases from disseminated RCC have been reported only infrequently. Pollheimer *et al*. [5] detected five new cases among 2,082 patients with RCC (0.2%). The mean (median, range) time to diagnosis of gastric metastases in the present case and those reported previously was 6 (4.7, 0.1 to 20) years; other studies showed even later appearance: 6.6 years (range: 2 years to 14 years) [7]. There are few studies (around 20) showing the association between gastric metastasis from RCC and other metastasis of the same tumor. Kim *et al*. in 2012 showed various case reports of gastric metastasis resection and other possible approaches to treating this disease [18].

The most frequent gastric tumor locations are in the body and gastric fundus, and single tumors predominate over multiple [3]; our patient had a solitary tumor at the proximal body on the greater curvature. The main symptoms, as described before, are upper GI bleeding (presenting as anemia, melena or upper hemorrhage) or gastric outlet obstruction [2].

There are various types of treatment such as embolization, epinephrine injection or systemic therapy (for example, octreotide and sorafenib) [12–17]. Even in patients without curative approach, surgery could be the elective treatment [7]. However, there are many issues involving the surgical approach, such as mortality, morbidity, the size and number of the metastatic disease, and patient condition. Thus, the laparoscopic approach appears to be a good alternative in these patients, particularly in palliative cases.

Examples of laparoscopic treatment of metastasis to the stomach in the literature include cases of melanoma [19] and choriocarcinoma [20], both treated by laparoscopic wedge resection (LWR) with good results and no perioperative mortality or morbidity. Laparoscopic resections for metastasis of RCC to other organs have also been described; an example is a distal pancreatectomy that showed great benefit [21].

The criteria for LWR in the stomach are tumor size up to 50 mm and tumor location on the lesser or greater curvature or on the anterior aspect of the gastric body [22]. Tumors near the pylorus and cardia are not suitable for LWR because of the technical difficulty and risk of complications [23]. Our patient, however, was eligible for the procedure.Laparoscopic wedge resection (LWR) is a simple technique and offers advantages of laparoscopic surgery, including a shorter hospital stay and early return to normal activity with minimal morbidity and mortality. The indications for this technique may be extended to the palliative resection of metastatic gastric tumors in selected patients.

## Consent

Written informed consent was obtained from the patient for publication of this Case Report and any accompanying images. A copy of the written consent is available for review by the Editor-in-Chief of this journal.

## Abbreviations

GI: gastrointestinal
ICU: intensive care unit
LWR: laparoscopic wedge resection
RCC: renal cell carcinoma.
